# What Socio-Economic and Political Factors Lead to Global Pesticide Dependence? A Critical Review from a Social Science Perspective

**DOI:** 10.3390/ijerph17218119

**Published:** 2020-11-03

**Authors:** Zhanping Hu

**Affiliations:** School of Humanities and Social Sciences, North China Electric Power University, Beijing 102206, China; huzhanping2006@163.com

**Keywords:** pesticide dependence, pesticide resistance, agricultural regime, farmers, agroecology

## Abstract

Dependence on chemical pesticides has become one of the most pressing challenges to global environmental sustainability and public health. Considerable regulatory efforts have been taken to mitigate pesticide dependence, which however has resulted in a prevalent ‘managerial failure’. Massive pesticide application has generated severe genetic resistance from pests, which has in turn further aggravated pesticide dependence and thus induced agrochemical industries to develop new pesticide varieties. This review proposes to look beyond the resistance-dependence nexus and presents a comprehensive discussion about global pesticide dependence in a social science perspective, i.e., revealing the socio-economic and political factors that reinforce pesticide dependence. These factors are classified into five intertwined themes: (1) agricultural regime, (2) social process of pesticide application, (3) economic analysis, (4) politics and governance, and (5) promotional failure of alternatives. It is found that pesticide dependence is not just a technological issue in the sphere of natural sciences, but more a human-made issue, with deep-seated socio-economic and political reasons. Addressing contemporary trap of global pesticide dependence entails a full acknowledgement and comprehension of the complex and intertwined factors. Furthermore, this review identifies two major explanatory approaches underlying the extant social science literature: a structuralist approach that stresses macro-level structures such as institutions, policies and paradigms, and an individualist approach that focuses on the decision-making of farmers at the micro level. This review recognizes the limitations of the two approaches and calls for transcending the duality. This study advocates a policy framework that emphasizes alignment and coordination from multi-dimensions, multi-actors and multi-scales. For future research, collaborations between natural and social scientists, and more integrated and interdisciplinary approaches should be strengthened.

## 1. Introduction

Pesticides are an array of agricultural innovations designed to protect plants from insects, pests and weeds. These manufactured chemicals have substantially benefited global agriculture and food security in multiple ways, but also brought potential hazards to environmental sustainability and public health [1,2,3]. Particularly, due to various reasons, overuse of pesticides has been long and widely practiced by farmers around the world, which has made the harmfulness even worse [4,5]. Despite with growing awareness of negative impacts from chemical pesticides, the quantity of pesticide production and consumption at the global level has kept increasing annually [6,7]. To mitigate the negative impacts, considerable efforts have been taken to reduce the use of chemical pesticides and promote more environmentally-friendly pest management methods worldwide, for example, Integrated Pest Management (IPM), and biopesticides, among others [3]. However, chemical pesticides are still the preferred choice for farmers [4,8,9,10]. This dilemma denotes a remarkable global phenomenon—pesticide dependence—which has raised long-standing challenges to global environmental management and public health [4,5,7,11,12].

Particularly, in terms of public health, pesticides presented a persistent and severe threat to human health, through multiple mechanisms, including dermal exposure, respiratory exposure, oral exposure, eye exposure, and contaminated food and water consumption, among others [13,14]. Pesticide users and general public both face the health threat from pesticides [13,14]. Extensive use of pesticides has been associated with a range of human diseases and disorders, including various cancers, respiratory disorders, diabetes, Parkinson’s disease, Leukemia, mental disorders, and neurological diseases, among others [13,14,15,16,17,18]. Besides, the persistent existence of highly hazardous pesticides on the market has been also associated with suicide in rural areas [19,20,21]. Researchers estimated that globally around 1.5 million people committed suicide by toxic pesticides from 1960-2018 [22]. Therefore, stepping off the track of pesticide dependence is vital to global public health.

Pesticide dependence denotes a process of intensification in pesticide use, manifest roughly in four aspects. At the micro/farmer level: (1) pesticide use intensity (pesticide use quantity per unit of land area) has increased persistently [12,23,24]; and (2) highly toxic or legally banned pesticides varieties have been continuously used by farmers [25]. At the macro level: (3) the scale of pesticide production and consumption has kept expanding at both national and global levels [26]; and (4) farmers have stuck to chemical pesticides despite the availability of alternatives [8,9,10]. In this article, global pesticide dependence principally denotes the persistent overuse of and reliance on chemical pesticides at both micro and macro levels. To be clear, the terms of pesticide dependence and pesticide overuse may be used interchangeably. Pesticide dependence can be considered as the origin of various pesticide-induced environmental, social, and health issues, as it is the process of ‘dependence’ that makes these issues persist, or locks farmers and agricultural systems into the pesticide trap [9,12,27,28].

The evolution of pesticide resistance by insect, pests and weeds has long been taken as the primary natural reason for pesticide dependence and various new varieties of pesticides have been constantly invented to combat the genetic evolutional process [25,29,30,31,32,33]. However, overemphasizing the natural and technological processes, although important indeed, may conceal more deep-seated socio-economic and political factors in human society, as pesticide dependence ‘is first and foremost a social question; technical fixes or regulatory changes will only acquire significance within the framework of serious social change’ ([34], p. 55). Hence, this review puts pesticides back into the social-political process context they are embedded in to explore the societal reasons of technological dependence. Most extant reviews regarding pesticide issues are based on an intradisciplinary approach and tend to treat pesticide issues as a technological and monodisciplinary problem. For example, there are reviews focusing on pesticide use and public health [27,35,36], alternatives to pesticides [10,37], and pesticide use and environmental ecotoxicology [28,38]. Although a wealth of studies attempt to seek the social-economic and political explanations for pesticide problems via a range of approaches, such as economics, policy sciences, sociology, anthropology, and political economy, among others, most of them either only focus on a limited number of factors or just mention the complex factors as research background. Studies focusing on comprehensive factors of pesticide dependence are relatively rare and patchy. It is of great necessity to do so because this can provide an advanced understanding of the pesticide problem in contemporary world and contribute to better environmental management and public health associated with pesticides. 

This review contributes to the literature in three aspects. First, it defines and examines pesticide dependence in an explicit manner, compared to most extant studies that ambiguously discuss the pesticide dependence issue. Second, the review presents a comprehensive synthesis of multiple factors leading to pesticide dependence. Third, it summarizes the extant studies into two major categories in terms of their underlying approaches: structuralist and individualist. The structuralist approach attempts to explain pesticide dependence in the structure dimension, and emphasizes the underlying institutions, policies, and paradigms that render pesticide dependence. The individualist approach, on the other hand, takes the decision-making of farmers as research foci, based on the assumption of rational individuals. This approach seeks to explain pesticide dependence through farmers’ ‘irrational’ decision-making and behavior on pesticide use. This review recognizes the limitations of the two approaches in understanding pesticide dependence, and advocates going beyond the dichotomy. 

In terms of methodology, this review takes a qualitative approach. Given the complexity and multidisciplinary feature of pesticide dependence studies, a rigorous systematic review approach, which often requires well-accepted and standardized variables in extant literature, seems inapplicable and also impossible for this review. As this review seeks to understand the multiplicity of the factors rendering global pesticide dependence, a theme-oriented approach was followed. In so doing, the author broadly read studies associated with pesticide dependence and gradually identified major themes, i.e., social, economic, and political themes. Guided by the themes, related literature was sought by various pathways. First, major databases (e.g., Google Scholar, Scopus, and Web of Science) were searched, using keywords including (but not limited to) pesticide dependence, pesticide overuse, pesticide treadmill, alternative to pesticides, IPM, pesticide politics, pesticide economics, and pesticide lock-in, among others (Appendix A). Second, a snowball strategy was employed to explore more related literature based on the bibliographies of searched journal articles and books. The snowball approach is important in this research because studies of pesticide dependence are extremely scattered across disciplines and often implicit. In the end, over a hundred references (including books and peer-reviewed articles in English) associated with pesticide dependence were collected to support this review. Of course, limitations of this method approach deserve to be mentioned. First, this review strategy lacks the rigor that a systematic review often possesses. Nonetheless, the acquired literature can sufficiently support the review because its aim is to identify themes instead of systematic examination of previous studies. Second, the notion of ‘global pesticide dependence’ in this review was used in a simplified manner, denoting an overall historical trend, which sheds inadequate light on the heterogeneity and variance among geographical locations and different types of pesticides. 

The paper proceeds as follows: Section 2 briefly presents an overview of pesticide dependence across the world, to show that the issue has existed and still exists at the global level. Then, it discusses pesticide resistance and its limitation in explaining pesticide dependence in the real world. Section 3 reviews various factors that explain pesticide dependence in the perspective of social science. Then, a summary of the review findings and discussions on policy implications and future research directions are presented in Section 4, following which Section 5 concludes the article. 

## 2. Setting the Scene: Global Pesticide Dependence and Pesticide Resistance

### 2.1. Global Pesticide Dependence

**Figure 1:**
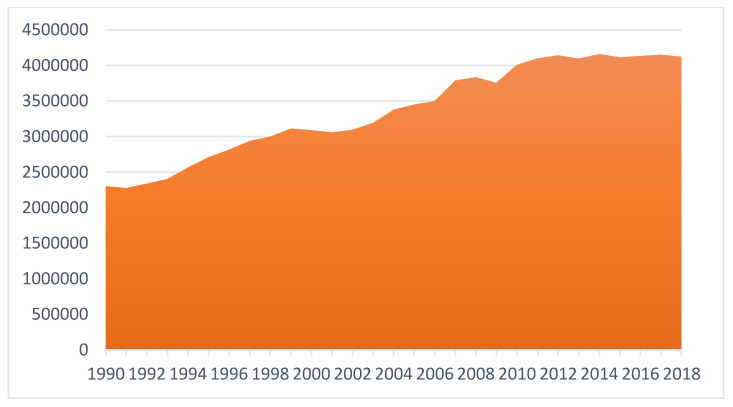
Global pesticide consumption trend from 1990–2018 (unit: tonnes). Data source: [39].

**Figure 2:**
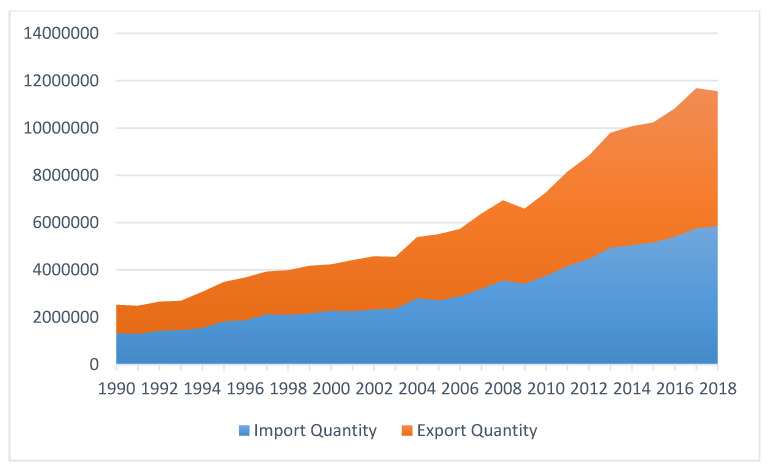
Global pesticide trade trend from 1990–2018 (unit: tonnes). Data source: [39].

**Figure 3:**
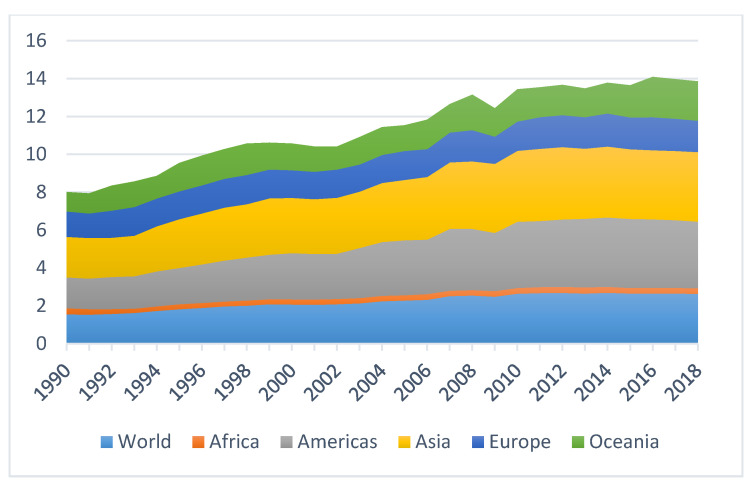
Global pesticide use intensity trend from 1990–2018 (measured by pesticide use per area of cropland, unit: kg/ha). Data source: [39].

Since the late 1930s when the organic synthetic pesticides were synthesized, pesticide consumption and varieties at the global level have continuously and dramatically increased, with the quantity expanding more than fifty-fold so far [6]. According to the latest data from Food and Agriculture Organization (FAO), the total pesticide consumption quantity of the world has risen from 2.3 million tonnes in 1990 to 4.1 million tonnes in 2018 [39] (Figure 1). Global pesticide trade has been also on the rise in a consistent fashion as shown by Figure 2. Moreover, pesticide use has been increasingly intensified across the world, although with unequal paces in different regions (Figure 3). 

In terms of geographical distribution, Asia ranks the first in pesticide consumption in the world at present, followed by North America. As for countries, China, USA, Brazil, and Argentina are the largest pesticide consumers in the world. China alone has been consuming almost half of the pesticides across the globe so far [10,40]. Similarly, pesticide use in Bangladesh increased at an annual rate of 10% between 1977 and 2009, with however a declining pesticide productivity at an annual rate of 8.6% in the same period [41].

Due to public concerns about environmental quality and human health, some developed countries, for instance, Denmark, France, Finland and Japan, have taken steps to reduce pesticide use intensity in recent years [39], but progress has been very slow [42]. Even with efforts to reduce application density, Ghimire and Woodward [43] found that developed countries with higher income per capita had been overusing pesticides. Meanwhile, pesticide application in most developing countries is still increasing [12,25,29,44]. These facts all imply that pesticide overuse is a prevailing and entrenched global phenomenon.

At the micro level, farmers apply pesticides in excess of recommended amounts across the world. Grovermann et al. [45] found that in horticulture in Thailand, 78–79% of pesticides applied were overused. A recent multinational study covering 1000 vegetable farmers from four countries in Southeast Asia showed that 100% of the sampled farmers in Vietnam, 73% in Cambodia and 59% in Laos overused pesticides [46]. Dasgupta et al. [47] found 47% of surveyed Bangladesh farmers overused averagely 3.4 kg pesticides per growing season. Chinese cotton farmers sprayed 11.8 kg/ha pesticides with optimal levels ranging from 0.4 kg/ha to 4.2 kg/ha, 3 to 30 times more than the optimal amount [48]. Two recent studies revealed that pesticide overuse widely existed in various types of Chinese farmers, including both vegetable and grain growers [49,50]. 

Additionally, as widely reported, farmers from many countries still tend to use pesticides containing active ingredients classified as highly or extremely hazardous by international organizations [30,51,52]. Moreover, alternatives such as IPM have been promoted worldwide since the 1970s, but the progress has been consistently slow and disappointing [2,8,9,10]. Chemical pesticides are still the first choice of global farmers, which signifies an evident process of pesticide lock-in. More worryingly, the world still faces enormous pressure to produce more agricultural products to satisfy the ever-growing population with gradual squeeze of arable land and scarcity of water resources; therefore, modern, convenient inputs such as pesticides still have tremendous momentum in the foreseeable future [7,44].

### 2.2. Pesticide Resistance as an Explanation

Pesticide resistance has been widely considered as one of the major causes of pesticide dependence [1,9,25,29,30,31,32,33,53,54,55,56]. Pesticide resistance represents a genetic mechanism whereby pests (including weeds) develop evolutional abilities to adapt to pesticides, and then the same variety and dosage of pesticides do not work on the adapted pests. It is essentially a process of genetic co-evolution in the ecological system [31], which suggests that pesticide resistance is a cause as well as a result of pesticide dependence.

In response to pesticide resistance, excessive concentration of pesticides have been applied and new varieties designed [1,2,33,54,57]. Consequently, not only are farmers caught on the never-ending race with pests’ evolution, but also the agrochemical industry has been motivated to promote new varieties of pesticides, thus further consolidating the pesticide dependence path. As Dyer ([31], pp. xii–xiii) wrote, “as new pesticides are produced and applied to kill unwanted organisms, the targeted pests adapt to each new chemical, which requires the development of new chemicals, which stimulates further adaption, and so on. For 60 years, farmers have become increasingly entrapped by what has been termed a ‘chemical dependency treadmill’’’. Since the invention of pesticides, pest and weed resistance has developed rapidly worldwide. So far, 954 species of pests, including 546 arthropods, 218 weeds and 190 plant pathogens have been recorded as species of pesticide resistance globally [58].

Undoubtedly, pesticide resistance has resulted in considerable overuse of pesticides, as well as severe toxicological damage to environment and public health [38,59]. This can increase the cost of pest control by nearly $40 million annually [57]. Genetically modified varieties have been developed to tackle pesticide resistance and have shown noticeable efficacy on pesticide reduction [44,48,60]. However, as shown by studies, albeit with adoption of crops that are resistant to insect pests, farmers still tend to use excessive dosage of pesticides [30,61]. As Pemsl et al. [61] commented, biotechnologies per se cannot address the pest problem, and researchers should change the strong belief that biotechnologies decide the future of pest control. No doubt, resistance from pests is a critical mechanism resulting in pesticide dependence worldwide; however, there are more deep-seated, often hidden, socio-economic and political reasons that have locked pesticides firmly along the process of agricultural production. Next section reviews in detail the multiple drivers of pesticide dependence in a social science perspective.

## 3. Reviewing the Social-Economic and Political Factors: Pesticide Dependence in a Social Science Perspective

The review discusses literature from a variety of social science disciplines, including economics, sociology, anthropology, politics, policy study, and human geography, among others. The factors contributing to global pesticide dependence fall in five broadly defined and intertwined themes: agricultural regime, social process of pesticide application, economic analysis, politics and governance, and promotional failure of alternatives. This section discusses each theme in detail. 

### 3.1. Agricultural Regime and Pesticide Dependence: from Pesticide Treadmill to Agricultural Treadmill

The first strand of studies regarding pesticide dependence explain how pesticides, as modern technologies, are locked into the whole agricultural modernization regime. Bosch [11] introduced the term of ‘pesticide treadmill’ referring to the race between pesticide resistance development and farmers’ pesticide application [2,33,62,63]. The pesticide treadmill was then enlarged to the debates of the whole agricultural regime. Clunies-Ross and Hildyard [64] critically examined industrial agriculture in developed countries with the term of ‘chemical treadmill’, which denoted an explanation of dramatic increase in the use of agrochemicals in the logic of industrial agriculture. They argued that the application of pesticides and fertilizers could improve yields impressively at the very start, and then yields went up and prices fell, and farmers who did not use chemicals would fall into bankruptcy due to the profit squeeze. Therefore, ‘those who adopted the new approach, however, stepped onto the chemical treadmill’ ([64], p. 61). 

Aware of treadmill theory’s neglect of historical situations, under the context of agricultural transformation in the UK, Ward [65] extended the concept of chemical treadmill to the whole capitalist agricultural regime, referring to ‘productivist treadmill’, in which farmers’ decision-making has increasingly been controlled by the agro-chemical industry. In developed countries, the productivist agriculture regime, taking shape after World War II and characterized by the exclusive emphasis on production quantity, has entrenched pesticides as an inseparable part [66]. More importantly, the ‘productivist agriculture’ regime was supported by strong political wills, government policies and public concerns about food supply, and therefore had dominated most western developed countries for decades [66].

In developing countries, the conventional agricultural development regime featured by Green Revolution technologies has been significantly connected with excessive pesticide adoption [62,67]. In this regime, to achieve agricultural modernization, the government encourage pesticides and other modern inputs through subsidies to boost land productivity [55]. Additionally, other Green Revolution technologies such as high-yielding varieties and chemical fertilizers are significantly dependent on the constant application of pesticides [9,41,68,69]. Meanwhile, traditional pest management technologies, which are often more environmentally-friendly, have been progressively replaced by synthetic pesticides [70,71]. Furthermore, the agricultural export strategy fueled by the Green Revolution in many developing countries, particularly in Latin America, has remarkably contributed to pesticide dependence [62,67,72]. For instance, Nicolls and Altieri [62] found that adoption of non-traditional export crops often drove farmers to excessively apply pesticides. Besides, Green Revolution has locked farmers’ livelihoods and labor process onto the pesticide treadmill [73]. In many cases, smallholders are aware of the risk of pesticide overuse, but have to apply them because their livelihoods rely on those chemicals [12,74,75,76]. Therefore, pesticide dependence derives from the dependence of smallholders on pesticide-driven livelihoods. Overall, pesticide dependence is an integral part of ‘productivist agriculture’ in developed countries [65,66], and Green Revolution-driven agriculture in developing countries [67,68]. In a recent study, Heldlund and colleagues [7] drew on global pesticide data (including both developed and developing countries) to examine the relationship between pesticide use and economic development. They found a consistent positive relationship between economic development and pesticide consumption amount over time, which suggests that the treadmill theory has been the dominant logic in both developed and developing countries and shown its power in explaining pesticide dependence. In line with this strand of studies, sustainable agricultural system has long been advocated worldwide as an alternative to agrochemical-dependent agricultural regime [3].

### 3.2. The Social Process of Pesticide Application and Dependence

#### 3.2.1. Agricultural Deskilling and Pesticide Dependence

Pesticides are a technological medium between farmers and the agro-ecosystem. Scholars have noted the deskilling process of farmers in interactions with modern agricultural technologies, including pesticides [63,77,78,79]. Vandeman [78] examined the relationship between pesticides commoditization and farmers’ pesticide application and found that commercial pesticides led farmers to fall into a dependent circle: the less farmers know about insect ecology, the more insecticides they use. Hence, this is a process of deskilling farmers’ agricultural practices, which as a result leads to dependence on pesticides. Stone [79], through distinguishing environmental learning and social learning of adopters in agricultural innovation diffusion, used ‘agricultural deskilling’ to interpret the cotton fad in Warangal, India, where farmers could not experiment and evaluate cotton varieties because of the accelerated rate of introduced technological changes.

According to Stone ([79], p. 73), agricultural deskilling is the ‘disruption of the balance between social and environmental learning that is instrumental in farm production’. The domination of social learning over environmental learning driven by external agricultural technologies drives farmers to rely on the information from social actors rather than observational and learning experiences from themselves. Two agricultural technologies--hybrid seeds and pesticides are prominently implicated in the deskilling process [79]. In sum, pesticides and other modern agricultural technologies deskill farmers, and in turn, the process of agricultural deskilling reinforces farmers’ dependence on pesticides. 

#### 3.2.2. Interactions between Farmers and External Social Actors

In practice, farmers apply pesticides based on associated information and risk perception; therefore, information provision and risk perception regarding pesticides are of particular importance to pesticide application. Pesticide information provision involves multiple actors, including governmental agricultural extension workers, pesticide suppliers, developers, farmers and their community peers. Interactions between these actors significantly influence pesticide application practices. Generally, pesticides are applied in low or normal dosage when farmers acquire accurate information [80]. However, due to poor quality of public service, the primary information provider in developing countries is frequently pesticide suppliers, who, as profit-pursuers, frequently amplify the dosage [30]. Jin et al. [80] found that in China, cotton farmers who used pesticide suppliers as their primary information provider tended to overuse pesticides. Additionally, trust plays a vital role in information provision in this process. Farmers tend to trust the information from family members, friends and governments, and show high level of distrust to pesticide suppliers. Consequently, given the generally high dosage recommended from pesticides suppliers, the distrust between farmers and suppliers may well further increase actual applied dosage [80]. Furthermore, instruction information on pesticide bottles is often thought too technical for farmers to understand [81,82]. The extreme diversity of pesticide varieties and fast update of new ones have rendered smallholders to rely on external information sources, which potentially and significantly contributes to pesticides overuse [79,80]. For instance, in China, there had been 627 active ingredients with 27,273 registered pesticides by 2012, and hundreds of new varieties come to market every year [80]. 

Relating to the discussion of agricultural deskilling, another form of learning process in agricultural decision-making was identified as ‘didactic learning’, which denotes the process that farmers’ agricultural knowledge is distorted by various stakeholders in agriculture, including governmental officials, external input providers, and NGOs, among others, through practices of ‘instruction, demonstration, exhortation, advertising, regulation, coercion, adulation, or shaming’ ([83], p. 10). Particularly, commercial didactic led by agrochemical corporations and retailers often takes the dominant form in rural world, rendering farmers to be caught on the pesticide treadmill [84].

Given the poor information acquisition in pesticide application, particularly in developing countries, farmers’ perception of pesticide risk is another potential factor instrumental in pesticide overuse. Especially, misperception of pesticides’ toxicity is one palpable driver of pesticide overuse (see Dasgupta et al. [47] for Bangladesh; Guivant [85] for Brazil). Also, researchers started to realize farmers’ diverse types of risk preferences and actions in pesticide use [30,61,73]. For instance, Liu and Huang [30] found that Chinese cotton farmers adopted risk-aversion instead of risk-seeking strategies, and therefore tended to overuse pesticides even when they planted insect-resistant Bt cotton to guarantee the output. They further commented that there were multiple reasons for farmers’ excessive usage of pesticides, and the prominent one was that the poorly-developed market system in China allowed many fake, low quality Bt cotton seeds and pesticides to permeate. This greatly damages farmers’ trust toward the products and forces them to use pesticides in excessive amount. In line with these findings, researchers proposed that education or training programmes could better inform farmers with accurate information regarding pesticide application and associated risks, and effectively reduce pesticide overuse [47,52,86]. Yet, researchers also casted criticisms on this ‘knowledge deficit model’ and proposed an acknowledgment of farmers’ abilities of understanding pesticide risks and a more participatory approach in information provision processes [12,73].

The social process of pesticide application at the farmer level is also closely associated with farmers’ pesticide use patterns in agricultural system [87]. The spatiotemporal patterns of farmers’ pesticide use have far-reaching and complicate impacts on agroecosystem (such as pollinators) and environmental toxicity [38,87]. Since farmers’ information and values regarding pesticide use are predominantly controlled by various external stakeholders [84,87], contemporary pesticide use patterns by farmers are generally misaligned with the normal functions of agroecosystem. Farmers’ information and awareness of the environmental impacts of pesticides was found an important driver to pesticide reduction [88]. In practice, however, farmers often face both information shortage and misinformation, with little awareness of environmental effects of pesticides. These factors may lead to pesticide overuse and misuse from framers and result in agroecological and environmental damage, which further creates ‘lock-in’ effect of pesticide dependence at the farmer level [87]. 

### 3.3. The Economics of Pesticide Dependence

Pesticide economics represents the dominant approach to understanding and addressing the pesticide issue. Thorough reviews of economics of pesticide issues have been well conducted in the literature [2,9,29,89]. This paper only centers on related studies regarding pesticide dependence.

#### 3.3.1. Economic Analysis of Pesticide Dependence

The economic analysis of pesticides fundamentally builds on the assumption that farmers are profit-maximizing actors and make rational decisions in technology application [9,45,89]. The major economic approach has been cost-benefit analysis, in which farmers make decisions based on the evaluation of the marginal product value and the economic costs of pesticides [45,90,91]. According to this principle, appropriate or optimal levels of pesticide use should be set at the equilibrium point where the marginal product values of pesticides equal to their marginal costs. In line with this analysis, it was found that the persistence of pesticide dependence derived from the fact that the marginal values produced by pesticides overtakes their marginal costs [89]. Consequently, the persistence of pesticide dependence can be attributed to the calculation that ‘pesticides appear to be more economically valuable than what could be concluded from pesticide expenditures’ ([89], p. 469). However, studies have also widely found that pesticide marginal product values often significantly exceeded their marginal costs in various geographical contexts, which clearly denotes a tendency of pesticide overuse [10,36,91]. It is noticeable that farmers often do not behave according to the principle of profit maximization as economic theories assume. To accurately evaluate the extent of pesticide use intensity, private optimum and social optimum levels of pesticide use which can be calculated by casting up external costs to purchase costs were developed [29,45]. Private optimum refers to the optimal levels of pesticide use from the perspective of farmers; and social optimum is the optimal levels of pesticide use that take pesticide negative externalities into account [45,92]. It was found that farmers’ private optimum levels of pesticide use were often well above social optimum levels because of the externalities of pesticides [45]. Moreover, economists have also computed that agricultural production will not be reduced even if certain amounts of pesticides (up to 30% in France) were cut off [2,93], clearly indicative of the existence of pesticide overuse in contemporary agriculture.

#### 3.3.2. Economic Instruments of Pesticide Reduction

A major concern of pesticide economics has been about how to reduce pesticide dependence by means of market incentives [2,29,89,93,94]. It has been widely argued that to effectively mitigate pesticide overuse, the negative externalities need to be internalized by farmers and pesticide producers [29,92]. Farmers pay relatively low costs for pesticides and reap all of the benefits in terms of immediate yield increases, while the long-term costs of damage to health and environment are borne by society at large. Market incentives, which refer to changing farmers’ and pesticide producers’ behaviors through economic stimulates, have increasingly been designed to reduce pesticide use and production. Related economic instruments include pesticide taxes, output subsidies, liability rules, revenue insurance, assignments of property rights, user-free tournaments, and tradable permits [29,90,93,95]. Economic instruments of pesticide reduction have mainly been promoted in developed countries such as EU countries [24] and the USA [29] and just started to apply in developing countries. In practice, these economic instruments have rendered varied effects on pesticide reduction, with disappointing outcomes in general, though. The most extensively adopted instrument is pesticide taxes, which however have been found to be consistently ineffective in reducing farmers’ pesticide use [2,29,45,90]. Moreover, Skevas et al. [94] examined the effects of four economic instruments on pesticide reduction in the context of Dutch agriculture and found that pesticide taxes, price penalties, and pesticides subsidies all failed to significantly reduce pesticide use, and only pesticide quotas had some effects. Many factors were attributed to the disappointing consequence. For instance, pesticide negative effects on environment are hardly observable at the farm level, making pesticide taxes on farm emission impracticable [29]. Meanwhile, farmers’ reluctance to respond to price or cost changes of pesticides reflects the characteristic of low demand elasticity, which ‘requires taxes so large that they become politically infeasible’ ([29], p. 300). Therefore, as studies have clearly shown, market mechanisms alone cannot effectively address pesticide overuse, and more comprehensive policy frameworks featured by multiple complementary instruments are expressly needed. Lastly, seeing from another angle, the poor performance of economic instruments has failed to reduce pesticide use, and therefore indirectly contributed to the persistence of pesticide dependence.

Overall, the economic examination of pesticide overuse has been heavily featured by neo-classical models which assume that farmers are profit-maximizing agents [9,90]. This simplified model, however, overlooks the complex ‘subjectivities’ of farmers in pesticide use, which suggests that farmers may use pesticides in different logics, instead of only the economic principle [12]. Meanwhile, ‘given the complexity of the pest control problem, simple economic optimization models could be unrepresentative or even misleading if used as a basis for policy recommendations’ ([90], p. 181). Therefore, ‘explicit account of farmer decision-making should be taken in environmental policy design’ because farmers may not necessarily act as a profit-maximizer ([90], p. 180). Lastly, researchers rightly reminded that pesticide reduction policies should not be considered separately, but in connection with the whole agricultural policy framework, including price supports, output limits, and quality standards, among others [29].

### 3.4. Politics, Governance and Pesticide Dependence

#### 3.4.1. Politics and Pesticide Dependence

Studies have shown that political institutions have significant correlations with pesticides use intensity. For instance, it was found that democratization could facilitate the reduction of pesticide use intensity in developing countries [96]. Democracy in general can create favorable political and social atmosphere for pro-environmental activities and policies. Consequently, environment-oriented policies can be more effectively formulated and implemented in democratic contexts [43]. The issue of pesticide dependence is embedded in the whole poor environmental regulation and policy framework; therefore, the quality and efficacy of overall environmental policies remain critical to pesticide reduction. This explains why some countries with high levels of democracy can successfully reduce pesticide intensity while others cannot. Also, as Pralle [97] argued in the context of Canadian pesticide politics, decentralization in the policy system may create stronger environmental regulations regarding pesticide use, because local people are empowered to enact environmental policies driven by public concerns.

In addition, there are special interest groups involved in pesticide politics, in which agrochemical industries often play particularly influential roles [11,23,98]. Driven by the incentive of capital accumulation, pesticide industries as powerful actors attempt to promote the sale and usage of pesticides by all means [99,100]. Meanwhile, they also maneuver to capture the regulators and hinder policies designed to reduce pesticide use, especially when associated regulations can effectively reduce pesticide use [23,98]. Not surprisingly, ‘pesticide industry interference’ is taken by experts from a body of countries as one of the major obstacles to promoting IPM in developing countries [101]. The effectiveness of pesticide industries’ efforts is closely tied to the regulation quality in the political sphere. It was found in the context of 24 OECD countries that poor bureaucratic quality, severe corruption, underdevelopment of legislative, judiciary and executive institutions and deeply vested special interest groups were major political drivers for ‘pesticide overload’ [23]. Obviously, pesticide industries have much more power in exerting influences and distorting related policies than ordinary people, especially when civil society is underdeveloped. As Wright ([68], p. xv) similarly argued, persistence of pesticide dependence has been based on ‘severe social imbalances that allow hazardous technologies to prevail because the pattern of rewards and penalties associated with the technology is rooted in the distribution of power in the society’. Moreover, it has been revealed decades ago that the involvement of pesticide industries in politics and scientific research, i.e., the ‘cynical conspiracy’, was the main reason that pesticides had been continuously manufactured, sold and promoted across the world [11].

Therefore, restriction of the influence of special interest groups in policy system could be an effective instrument for mitigating pesticide dependence. To either realize food self-supply or improve public hygiene, there have been strong political wills from the government encouraging pesticide use. Many countries that face tight pressures of growing population tend to loosen the regulation on pesticides and thus implicitly promote pesticide application, which has been an influential political driver for pesticide dependence, especially in developing countries, such as China, Vietnam [102], and Thailand [45]. At the global level, Hough [103,104] found that weak political strength at the international level could not produce powerful regulation and therefore resulted in poor global pesticide governance. Also, global civil society has not been powerful enough to effectively regulate the production and application of pesticides [104]. Although global environmental organizations have managed to promote various international regulation frameworks on pesticides reduction and banning, for instances, the Rotterdam Convention in 1998 and the Persistent Organic Pollutants Treaty in 2001. These frameworks however were found extremely difficult to translate into domestic practices [105]. One reason lies in that there are no international mandatory forces that can stringently implement the international regulations at the domestic level [104]. Additionally, using the case of Honduras, Jansen [105] amply demonstrated that in the regulation transfer process, agronomists who dominated the domestic policymaking often concentrated on their technical knowledge, and rarely took the dynamic process of how pesticides were used in everyday life into account. Obviously, agronomists have mightier power and influence than the public and smallholder farmers in the domestic policymaking, and the imbalance of power and the absence of smallholders in the policymaking process have blocked the transfer of international regulations into the domestic sphere [105]. Using the Californian strawberry industry as a case study, Mayfield and Norman [106] illustrated the process of pesticide transition from an internationally banned variety (methyl bromide) to an environmentally-friendly alternative (iodomethane) under the Montreal Protocol. They found that the strawberry industry in California had successfully manipulated the transfer process of Montreal Protocol to domestic policies and extended the use period of methyl bromide through various political and economic means, such as lobbying policy makers.

Moreover, transnational commercial organizations have exercised significant influences on developing countries because many of these countries are heavily dependent on foreign investments due to various political-economic reasons [69]. It was found that pesticide use intensity in developing countries was positively correlated with foreign investment dependence [43,69,96]. The governments of these developing countries cannot effectively initiate and implement the policies oriented to pesticides reduction, given severe structural dependence on foreign capital [69]. Therefore, the pesticide dependence in the domestic sphere of developing countries derives from the deep-seated socio-economic and political dependence on developed countries at the international level.

#### 3.4.2. Pesticide Management: A Governance Perspective

Pesticide dependence was conceptualized as a market failure in need of governmental interventions [9,61]. However, in practice, related governance measures often fail to do so, demonstrating a ‘managerial failure’ in pesticide regulation [23]. Researchers have shown worldwide that poor regulations and insufficient public services lead to pesticides overuse [47,80,102,103,104]. Poor governmental policy frameworks regarding environment protection as a whole and pesticide use in particular result in pesticide overuse, such as implementation of the legislation and control of pesticide residues, funding of research and extension, among others [102]. Extension services from governments in developing countries are generally underdeveloped, so that pesticide information provision to farmers is poor [30,102].

Consequently, information source is dominated by pesticide suppliers, which is a significant element leading to pesticide overuse, as discussed in the above sections. In China, agricultural extension workers’ salaries are often linked with pesticides sale profits and therefore they are encouraged to promote pesticide overuse rather than appropriate use [48]. Other regulatory instruments include pesticide bans, use restrictions and control on overall quantity; however, these instruments have been largely ineffective [29]. For instance, pesticide bans have been largely ineffective in reducing pesticide use, and often result in higher output prices which consequently favor pesticide producers and harms customers and farmers [29]. In many developing countries, a ban on highly toxic pesticides has not been put into policymaking agenda [105]. High levels of transaction costs of pesticide reduction policies in developing countries also hinder the efficacy of these policies [100]. Furthermore, weak regulations in domestic markets in developing countries often lead to severe pesticide overuse because the agricultural products for domestic markets are not stringently examined in terms of pesticide residues [51,107]. Overall, the consistent managerial failure of pesticide regulation across the globe has reflected the complexity of pesticide dependence on the one hand and contributed to the persistence of pesticide dependence on the other.

### 3.5. The ‘Promotional Failure’ of Pesticide Alternatives

Pesticide dependence would not have persisted for such a long time if alternatives could be successfully implemented. An important reason for the persistence of pesticide dependence is exactly that there exist immense difficulties for alternatives such as IPM to substitute pesticides in real socio-economic and political contexts, resulting in a ‘promotional failure’ of pesticide alternatives [10]. Researchers have agreed that pesticides have been firmly locked into the socio-economic and political path of modern society through various mechanisms, leaving little room for alternatives to develop [8,9,10,93,108,109,110]. In the competition between two types of technologies, IPM methods have been particularly disadvantageous to chemical pesticides. Cowan and Gunby [8] presented an empirical study of technological competition between chemical pesticides and IPM via illustrating the properties of technology choice—‘positive feedback, self-reinforcing mechanisms, and the importance of relatively minor events in pushing the system in a particular direction’ ([8], p. 522). They revealed that increasing returns from pesticide application, high Research and Development (R&D) costs for chemical control development, markets locked into pesticides, high switching costs from chemical pesticides to IPM, the knowledge intensive characteristics and uncertain effects of IPM all made farmers unable to overcome the technology inertia, and therefore locked into the pesticide application path, although IPM might be superior to the chemical control strategy in many aspects. Through analyzing the characteristics of IPM and pesticides respectively, Woff and Recke [108] found that in Ghana chemical pesticides were entrenched and squeezed out the application of IPM through individual learning, level of implementation, network externalities, interest rate, and uncertainty reduction. IPM represents a long-term, systematic approach to pest control, which however has been often downplayed in a modernizing society which values short-term growth and conventional development [10,67].

Moreover, the institutional environment and contemporary policy frameworks are not supportive to the promotion of IPM, and chemical industry may well utilize IPM as an instrument to sell chemical pesticides [2,10]. Consequently, IPM has been poorly promoted at the global level, with low adoption rates [101,109,111]. Ironically, researchers found that even the adoption of alternative technologies also led to pesticide overuse, due to farmers’ strong risk-aversion strategies in agricultural production and poor governmental supports [30,61,74,112]. For instance, Togbé et al. [112] revealed the technological and institutional constraints of an alternative pest control strategy which could potentially save pesticides use from 44% to 54% in Benin. They found that technological complexity, poor educational performance of farmers, low levels of extension service, lack of financial and political support, powerlessness of farmers, weak farmer-based organization, and incoordination between farmers and input suppliers all contributed to the poor performance or abandonment of the alternative strategy, and farmers returned to conventional pesticides spray in the end. Overall, these studies convey a clear message that contemporary social-technical and political structures favor chemical pesticides rather than the alternatives.

## 4. Summary and Implications for Policy and Future Research Agenda

Pesticide dependence has brought and may well continue to bring enormous and long-standing challenges to environmental management and human health. Genetic resistance from pests is an important reason for pesticide dependence; nonetheless, this research has shown that pesticide dependence is not only a natural process inherited from genetic evolution, but more a societal issue created by human society, involving factors from multi-dimensions, multi-actors, and multi-scales. This section first summarizes the major findings drawn from the literature, and then discusses relevant policy implications to mitigate pesticide dependence. Lastly, this section points out future research directions.

### 4.1. Summary of Socio-Economic and Political Drivers of Global Pesticide Dependence

The major findings from the literature can be briefly summarized as follows:

- Pesticide dependence is not only a technological issue as pesticide resistance thesis indicates. Comprehensive and deep-seated socio-economic and political factors have resulted in and perpetuated pesticide dependence.
- Pesticide dependence is an integral part of modern agricultural regime in both developed and developing countries. Mitigation of pesticide dependence rests not only in efforts from the specific technological realm, but also in changes of modern agricultural regime.
- At the micro level, facing fast changes in agricultural technologies, farmers, especially smallholders in developing countries, have lost the autonomy of using technologies, and been deskilled by rapidly changing technologies. The deskilling process has resulted in further dependence on not only chemical pesticides, but also the whole modern agricultural technological regime.
- Farmers’ pesticide application is embedded in specific socio-economic and political contexts, whereby various actors from governments, markets and communities influence farmers’ decision-making. Ineffective and insufficient communications between farmers and other actors lead to pesticide overuse.
- Farmers have different perceptions on pesticide risks and adopt different risk strategies. Inaccurate perception of pesticide risks and strong risk-aversion preferences by farmers often results in significant pesticide overuse.
- Pesticide dependence cannot be addressed solely through market incentives. Many economic instruments have failed to reduce pesticide use worldwide. More integrated approaches are needed.
- Political factors have played vital roles in pesticide dependence. Democracy, bureaucratic quality, political influences of pesticide industries, and civil society all have strong correlations with pesticide dependence at both domestic and global levels.
- From the management perspective, pesticide governance has been largely ineffective both at the domestic and international levels. Poor public services for pesticide application and the transfer failure of international pesticide regulation into domestic policies contribute to pesticide dependence.
- Pesticide alternatives have been promoted globally, but with disappointing results in comparison with the continuous expansion of chemical pesticides. Multiple reasons at both micro and macro levels have contributed to the path dependence of pesticides and the socio-technical and political exclusion of the alternatives.
- Given the highly diverse drivers of pesticide dependence, more radical, comprehensive and coordinated approaches need to be considered.

### 4.2. Policy Implications for Mitigation of Pesticide Dependence

This review highlights that different disciplines approach pesticide dependence from different perspectives and studies have often been conducted in a compartmentalized manner. It also reveals that mitigation of pesticide dependence needs multi-dimensional efforts from a full range of disciplinary spheres. As well documented, policies from a singular perspective can hardly address this intricate issue [2,9,23,29,87]. Importantly, technological advances alone cannot solve pesticide dependence, and coordinated efforts from various related aspects are of strong necessity. As Vanloqueren and Baret [109] and Togbé et al. [112] demonstrated, given that alternative technologies are confronted with enormous socio-economic, institutional and political constraints, to remove them, alignment from different dimensions at different scales is particularly critical. More specific intervention implications are discussed as follows, informed by the findings from the literature.

At the farmer level, a wealth of studies proposed education or training programmes for farmers to enhance their knowledge levels and provide accurate evaluation of pesticide risks [47,52,86,102]. However, quite a few studies contended that this ‘knowledge deficit model’ was largely invalid and weak in explanatory power because even the farmers that have sufficient education and knowledge still tend to overuse pesticides [12,73]. The reason lies in that farmers’ decision-making is not only determined by knowledge and information, but also influenced by the political-economic conditions, livelihoods, and labor processes [12,73,113]. Shattuck [113] found recently in the context of Northern Laos that farmers’ knowledge regarding pesticide risks is neither ignorant nor well informed, but contextual and partial. Therefore, education or training programmes must work with broader and more comprehensive socio-economic and political reforms or policy packages, which can ameliorate the whole political-economic constraints that farmers face, rather than just center on pesticide use. Nonetheless, this does not indicate that the information-educational model should be abandoned. Farmers are the major pesticide exposure social group and face enormous health threats due to close contact with pesticides [13,27,28]. Farmers’ information and knowledge regarding the health effects of pesticides serves as both a driver for pesticide dependence and a pathway to mitigating it [42,87,114]. Information and education regarding the health impacts of pesticide is still necessary for public health. In addition, given the disappointing efficacy of education and training programmes in practice, which are often conducted in a top-down didactic manner [83], more interactive, communicative and participatory approaches need to be crafted. This can bridge the gap between awareness and action/behavior, empowering grassroots actors to sufficiently participate in the process of programme design and implementation [12].

At the agricultural regime level, alternative paradigms to industrial or productivism agriculture have emerged in recent decades and played significant roles in facilitating sustainable transition. While most countries, particular developing countries, are still dominated by industrial agriculture regime, in Europe and Australia, policy frameworks and social practices regarding agriculture and rural space have embarked on transitions towards post-productivism or multifunctionality, with remarkable emphasis on environmental sustainability of agriculture and pluriactivity of rural socio-economy [66,115]. Agroecology represents another strand of efforts to resist the dominance of industrial agriculture and radically transform contemporary external-inputs-dependent and profit oriented agriculture into sustainable and just paradigm [116,117,118]. Agroecology has developed into a holistic research-practice framework that integrates ‘research, education, action and change that brings sustainability to all parts of the food system: ecological, economic, and social’, guided by ecological principles ([117], p. 599). Under the umbrella of agroecology approach, multiple farming practices or patterns have emerged across the world to reduce or eliminate external inputs such as pesticides and fertilizers in farming process and mobilize to use local resources [118,119]. Typical agroecology-oriented farming patterns include (but not limited to) precision farming, farming economically, organic farming, peasant-style farming, IPM, community support agriculture, nested markets, and zero-budget natural farming, among others. Recent studies convincingly show the economic potential and social resilience of agroecology-oriented farming patterns across the world [120]. In practice, agroecology still faces enormous challenges in scaling up, while given its vast potential on reconciling the tensions of food security, environmental sustainability, and social justice, it is a model worth proactively pursuing.

Importantly, the transformation of agricultural regime must be aligned with the change of rural communities, as agriculture is embedded in the socio-economic and political contexts of rural society. Therefore, sustainable agriculture must be built together with sustainable rural communities [115,121]. Mechanisms that are conducive to sustainable rural development should be explored and taken into policy framework. Structures and forces at macro level (i.e., (counter-)urbanization, modernization, globalization, and environmentalism) matter undoubtedly, but real change ultimately relies on local actors, who are the core agent ‘translating the various forces impacting on local communities into positive outcomes for the community’ ([115], p. 25). Therefore, enabling and inclusive policy frameworks are pivotal to promote sustainable agriculture and rural communities.

At the government policy level, this review points out that political factors significantly impact pesticide dependence worldwide. It might be difficult to take political reform into account because policies themselves are forged in particular political settings. However, it does suggest the importance of democracy, bureaucratic quality, and civil society towards environmental management. Therefore, policies or regulations aiming to encourage public participation, improve bureaucratic efficacy, reduce corruption, and foster civil society are urgently needed and highly recommended. This paper has also demonstrated geographical imbalance of pesticide dependence across the world. Although pesticide dependence is prevalent in most countries, some countries do show higher levels of severity than others, and some developed countries have done observable progresses in reducing pesticide use intensity in recent decades [44]. The most serious scenario happens in developing countries such as China, Vietnam, and Costa Rica, among others, and shared features in these countries include under-developed market systems, poor public services, food security pressures, and disadvantageous positions in global food chain. Therefore, policies designed to improve market functions and public services, although may not change the whole situation, may well help mitigate pesticide dependence.

At the global level, this research has stressed that there are large gaps between international pesticide regulations and domestic policy frameworks [104,105]. The binding force of international regulations has remained weak and ineffective to individual countries due to various political and institutional reasons. Yet, Galt [51] found an significant shift of pesticide complex at the global level, and that with stringent international market regulation and implementations, pesticide overuse has been effectively controlled in international agro-food market but still evidently remained in domestic markets where regulation and implementation are poorly operated. This indicates that governments of developing countries which produce both for international and domestic markets should further strengthen and strictly implement pesticide-related regulations for domestic markets to mitigate pesticide dependence. Overall, given that pesticide dependence is driven by multi-faceted factors, the most pivotal policy implication from this review is that policy reform in any particular sphere can never address the problem, but coordinated efforts from multiple actors, dimensions and geographical levels need to be urgently put into policy agenda. As Ríos-González and colleagues rightly commented, addressing pesticide dependence issue ‘requires multiple actions at different levels, ranging from the intra and interpersonal level to market and political structures…’ ([73], p. 51).

### 4.3. Future Research Agenda

This review calls for multi-disciplinary approaches in pesticide studies. The literature has well documented the complexity of pesticide dependence. However, the majority of related studies were separately conducted in particular disciplines. Natural scientists predominately focus on how to address pesticide resistance and other evolutional processes and develop new seed and pesticide varieties. Social scientists have approached pesticide dependence through diverse disciplinary perspectives and produced varied explanations and policy suggestions. Notwithstanding, it has been explicitly shown in the above sections that any single approach can never sufficiently explain and effectively address pesticide dependence. There is a strong need for multi-disciplinary research which combines insights from both natural and social scientists [74,87]. Pesticide application is an extremely complicated process in practice, and the effects on productivity, environment and human health are even more intricate and uncertain [38,45,59,90], which is a serious challenge for social scientists without related backgrounds in investigation. Similar challenges exist too for natural scientists in dealing with socio-economic, cultural and political factors in pesticide studies. Hence, collaborative research between different disciplines with humility and honesty should be strongly encouraged.

Overall, two fundamental approaches have underlain current literature of pesticide dependence. One is structuralist approach which underscores the external structural forces in determining farmers’ decision-marking in pesticide application. Studies on political-economic factors and agricultural regimes generally follow this approach. In the analysis of structuralist studies, the imperfection of various ‘structures’ at the macro level transmits into pesticide dependence at the micro level. This approach is instrumental in understanding deep-seated structural factors contributing to pesticide dependence, but may downplay the grassroots agency, and view farmers as passive actors subject to the external political-economic conditions.

The other underlying approach is individualist approach that takes farmers as rational ‘homo economicus’, and self-responsible actors [12]. Economic studies often adopt this approach as the starting point [9,45,47]. This strand of studies looks into farmers’ decision-marking at the micro level, significantly supplementing the shortfall of the structuralist approach. Yet, this approach often takes farmers as single-dimensional rational human being, overlooking the other existent and influential elements and farmers’ heterogeneous subjectivities. As Galt [12] amply argued in the context of pesticide use in Costa Rica, farmers possess multiple subjectivities in pesticide use. Similarly, Bakker et al. [122] also found farmers’ pesticide application practices are driven by multiple motivations, including moral considerations, social influences and sense of self-realization, rather than just perform as ‘homo agricola economicus’.

Therefore, the two approaches both are reductionist in explaining pesticide dependence. The situation of pesticide dependence in specific context is formed by both structural and behavioral elements. Studies of pesticide dependence should overcome the shortfalls of the two dominant approaches, and an integrated approach is needed in the future, in which both farmers’ heterogeneous subjectivities and socio-economic and political conditions that they are situated in should be taken account for. Lastly, as well documented, farmers have been aware of the harmfulness of pesticides but still tend to overuse them. The obvious gap between awareness and reduction actions is the most intriguing part of pesticide dependence studies at the micro level, which however has been greatly under researched in literature. Given the critical importance of farmers in the issue of pesticide dependence, studies focusing on this theme should be strengthened in the future.

## 5. Conclusions

Pesticide dependence has long been one of the major threats to environment and public health. It has shown a manifest tendency of pesticide dependence across the world, which may well continue in the foreseeable future. It is of particular necessity to understand the underlying reasons for this haunting global issue. Technological-centered approaches including pesticide resistance and new alternatives have failed to sufficiently explain and effectively address the long-standing pesticide dependence. This review paper illustrates that there are more complex and deep-seated socio-economic and political reasons for the persistence of pesticide dependence across the world. These factors exert influences at multi-sales, ranging from the farmer level to the international level. In consequence, policy frameworks based on any single discipline and any single approach have been largely ineffective. At the management level, there is a strong need of alignment and coordinated efforts combining multiple related actors across multiple socio-economic and political spheres, and geographical scales. For future research agenda, collaborations between multiple disciplines are strongly recommended. In addition, an integrated approach should be further explored to overcome the shortfalls of existing approaches. Research on farmers’ decision making and actions should be encouraged given the critical importance of farmers in the process of pesticide dependence.

## Appendix A. Major Sources and Journals Consulted for this Review

This review took a mixed literature searching strategy to scope related studies. The major sources and journals that this review consulted are listed in the following table.

**Table A1:** The lists of the major literature sources and journals consulted by the author.

	Source Name	Major Search Strings
Major literature sources (not exhaustive)	Web of science	Pesticide + use/overuse Pesticide + intensity Pesticide + dependence/path dependence/lock in Pesticide + resistance/treadmill Pesticide + economics/economic analysis Pesticide + politics/power/management Pesticide + environmental management Pesticide + sociology/environmental sociology Pesticide + health Pesticide + alternatives/IPM/agroecology
	Google Scholar	
	Scopus	
	PubMed	
Major journals (not exhaustive)	*Science, Nature, Proceedings of the National Academy of Sciences of the United States of America, Agronomy for Sustainable Development, Science of the Total Environment, Journal of Environmental Management, Global Environmental Change, Crop Protection, Ecological Economics, Environmental Research, Agricultural Economics, The American Journal of Agricultural Economics, Economic Journal, Journal of Rural Studies, Journal of Peasant Studies, Current Anthropology, Journal of Political Ecology, Human Ecology, Ecology & Society, Agriculture and Human Values, Food Policy, International Journal of Environmental Research and Public Health, Rural Sociology.*	
